# Supplementary material for: Pharmacokinetics and Novel Metabolite Identification of Tartary Buckwheat Extracts in Beagle Dogs Following Co-Administration with Ethanol
Source: Pharmaceutics. 2019 Oct 12;11(10):525. doi: 10.3390/pharmaceutics11100525 (PMC6836259; doi:10.3390/pharmaceutics11100525)
Supplement: Supplementary file 1 [file pharmaceutics-11-00525-s001.pdf]

pharmaceutics

Supplementary Materials: Pharmacokinetics and Novel Metabolite Identification of Tartary Buckwheat Extracts in Beagle Dogs Following Co-Administration with Ethanol

Yuancai Liu, Jun Gan, Wanyu Liu, Xin Zhang, Jian Xu, Yue Wu, Yuejun Yang, Luqin Si, Gao Li and Jiangeng Huang

Table S1. List of ion pair parameters, declustering potential (DP), collision energy (CE), cell exit potential (CXP) for quercetin, kaempferol and d_3-quercetin (IS).

Analyte	Q1 Mass(Da)	Q3 Mass(Da)	DP(V)	CE(eV)	CXP(V)
quercetin	301.0	150.9	−100	−28	−7
kaempferol	284.9	92.8	−115	−48	−5
d_3-quercetin(IS)	304.0	150.9	−100	−28	−7

Table S2. The linearity of quercetin in beagle dog plasma measured by LC-MS/MS.

Nominal Concentration (ng/mL)		Batch 1		Batch 2		Batch 3	
		Measured Concentration (ng/mL)	Accuracy (RE, %)	Measured Concentration (ng/mL)	Accuracy (RE, %)	Measured Concentration (ng/mL)	Accuracy (RE, %)
0.5		0.487	−2.60	0.510	2.00	0.518	4.00
1		1.05	5.00	0.949	−5.10	0.920	−8.00
5		5.09	2.00	5.21	4.00	5.15	3.00
50		50.9	2.00	51.2	2.00	50.6	1.00
200		197	−1.70	208	4.00	204	2.00
500		537	7.00	495	−1.00	498	−0.400
900		837	−7.00	888	−1.40	895	−0.600
1000		956	−4.40	950	−5.00	988	−1.20
Parameter	a	0.00848		0.00826		0.00832	
	b	0.00563		0.00810		0.00556	
	r	0.999		0.999		0.999	

Table S3. The linearity of kaempferol in beagle dog plasma measured by LC-MS/MS.

Nominal Concentration (ng/mL)		Batch 1		Batch 2		Batch 3	
		Measured Concentration (ng/mL)	Accuracy (RE, %)	Measured Concentration (ng/mL)	Accuracy (RE, %)	Measured Concentration (ng/mL)	Accuracy (RE, %)
0.5		0.468	−6.40	0.472	−5.50	0.506	1.00
1		1.12	12.0	1.11	11.0	1.00	0
5		5.09	2.00	5.05	1.00	4.45	−10.9
50		49.0	−2.00	50.1	0	46.0	−8.00
200		192	−4.00	193	−3.50	200	−0.20
500		544	9.00	484	−3.20	520	4.00
900		829	−7.90	912	1.00	975	8.00
1000		972	−2.80	988	−1.20	1060	6.00
Parameter	a	0.00318		0.00292		0.00322	
	b	0.000480		0.000556		0.000669	
	r	0.997		0.999		0.998	

Table S4. Intra-day and inter-day accuracy and precision of quercetin at all QC levels.

Nominal Concentration (ng/mL)	Intra-day (n = 6)				Inter-day (n = 18)			
	Measured Concentration (ng/mL)		Accuracy (RE, %)	Precision (RSD, %)	Measured Concentration (ng/mL)		Accuracy (RE, %)	Precision (RSD, %)
	Mean	SD			Mean	SD		
0.5	0.510	0.0421	2.07	8.25	0.505	0.0461	0.900	9.13
1.5	1.58	0.0598	5.44	3.78	1.55	0.0946	3.07	6.12
30	31.7	0.869	5.56	2.74	32.1	1.02	7.06	3.16
750	757	9.11	0.956	1.20	756	18.8	0.852	2.48

Table S5. Intra-day and inter-day accuracy and precision of kaempferol at all QC level.

Nominal Concentration (ng/mL)	Intra-day (*n* = 6)				Inter-day (*n* = 18)			
	Measured Concentration (ng/mL)		Accuracy (RE, %)	Precision (RSD, %)	Measured Concentration (ng/mL)		Accuracy (RE, %)	Precision (RSD, %)
	Mean	SD			Mean	SD		
0.5	0.529	0.0323	5.84	6.10	0.546	0.0260	9.15	4.77
1.5	1.64	0.0657	9.00	4.02	1.64	0.0975	9.48	5.94
30	31.7	1.12	5.56	3.54	31.6	1.96	5.31	6.20
750	809	20.0	7.82	2.48	799	39.1	6.53	4.89

Table S6. Matrix effect and extraction recovery of quercetin, kaempferol and IS in dog plasma at three QC levels (*n* = 6).

Analyte	Nominal Concentration (ng/mL)	Matrix Effect (%)			Extraction Recovery (%)		
		Mean (%)	SD (%)	RSD (%)	Mean (%)	SD (%)	RSD (%)
Quercetin	1.5	102.52	6.29	6.14	86.12	2.39	2.78
	30	105.15	3.03	2.88	68.40	4.89	7.15
	750	101.16	1.70	1.68	69.33	7.31	10.54
Kaempferol	1.5	95.67	6.24	6.52	84.96	2.85	3.35
	30	96.97	2.59	2.67	68.76	5.06	7.36
	750	93.89	3.18	3.38	71.23	3.96	5.55
IS	100	105.40	2.71	2.57	75.22	5.70	7.57

Table S7. The dilution reliability of quercetin and kaempferol.

Nominal Concentration (ng/mL)	Quercetin		Kaempferol	
	Accuracy (RE, %)	RSD (%)	Accuracy (RE, %)	RSD (%)
4000	2.00	2.94	0.00	4.26
4000	−4.70		−6.50	
4000	0.00		−6.60	
4000	−4.80		−8.10	
4000	1.00		2.00	
4000	-2.70		-5.30	

Table S8. Stability of quercetin in dog plasma under various conditions.

Stability	Sample Condition	Nominal Concentration (ng/mL)	Measured Concentration (ng/mL)		Accuracy (RE, %)	Precision (RSD, %)
			Mean	SD		
Freeze-thaw cycles	4 cycles	1.5	1.3	0.01	−13.67	0.55
		30	30.3	1.06	1	3.49
		750	706	25.7	−5.82	3.63
Short term	Room temperature (24 h)	1.5	1.55	0.18	3.11	11.66
		30	32	1.61	6.67	5.03
		750	755	12.5	0.62	1.66
Processed sample	Auto-sampler (4 °C, 24 h)	1.5	1.6	0.11	6.67	7.1
		30	32.7	1.29	8.89	3.94
		750	733	34.6	−2.27	4.72
Long term	30 days at −80 °C	1.5	1.6	0.1	6.67	6.53
		30	30.2	1.25	0.67	4.15
		750	719	2.65	−4.13	0.37

Table S9. Stability of kaempferol in dog plasma under various conditions.

Stability	Sample Condition	Nominal Concentration (ng/mL)	Measured Concentration (ng/mL)		Accuracy (RE, %)	Precision (RSD, %)
			Mean	SD		
Freeze-thaw cycles	4 cycles	1.5	1.67	0.03	11.11	1.93
		30	28.3	1.12	−5.56	3.94
		750	745	9.64	−0.67	1.29
Short term	Room temperature (24 h)	1.5	1.65	0.12	9.78	7.25
		30	27.8	2.55	−7.33	9.18
		750	698	28.3	−6.93	4.05
Processed sample	Auto-sampler (4 °C, 24 h)	1.5	1.42	0.15	−5.33	10.23
		30	26.6	1.01	−11.44	3.79
		750	709	34.3	−5.47	4.83
Long term	30 days at −80 °C	1.5	1.53	0.08	1.78	4.92
		30	26.5	0.89	−11.67	3.35
		750	658	9.02	−12.31	1.37

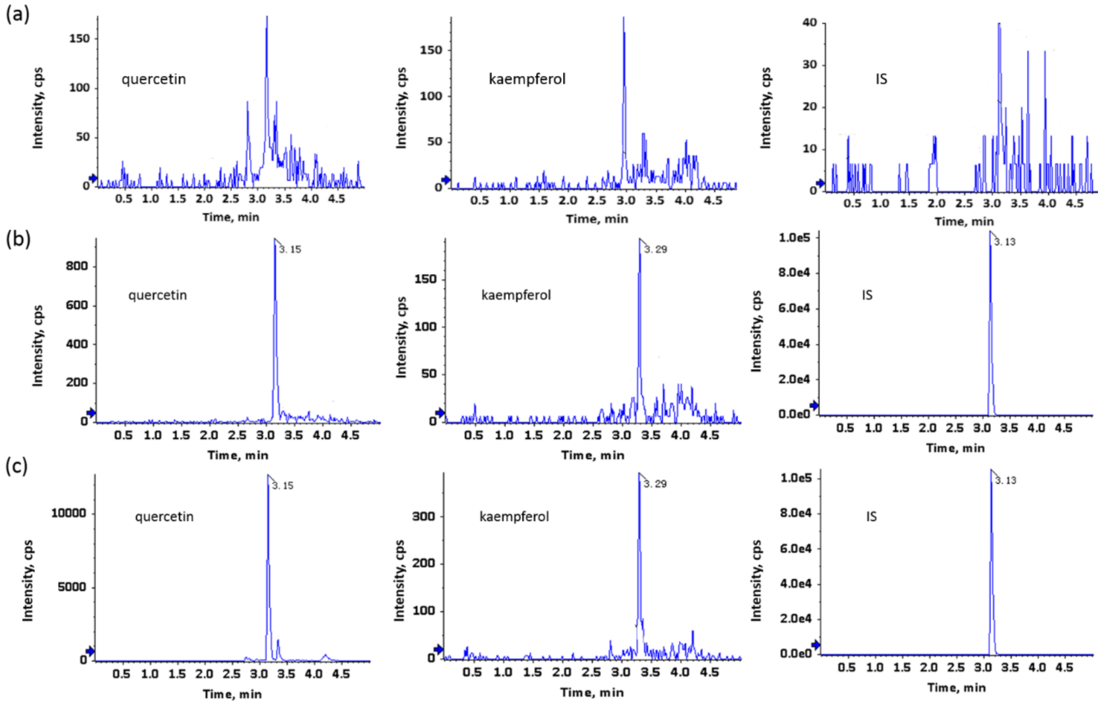

Figure S1. Typical chromatograms of quercetin, kaempferol and d3-quercetin (IS) in dog plasma: (**a**) blank dog plasma, (**b**) blank plasma spiked with analytes and IS at LLOQ level, (**c**) plasma sample obtained from beagle dog after oral administration of tartary buckwheat extract.

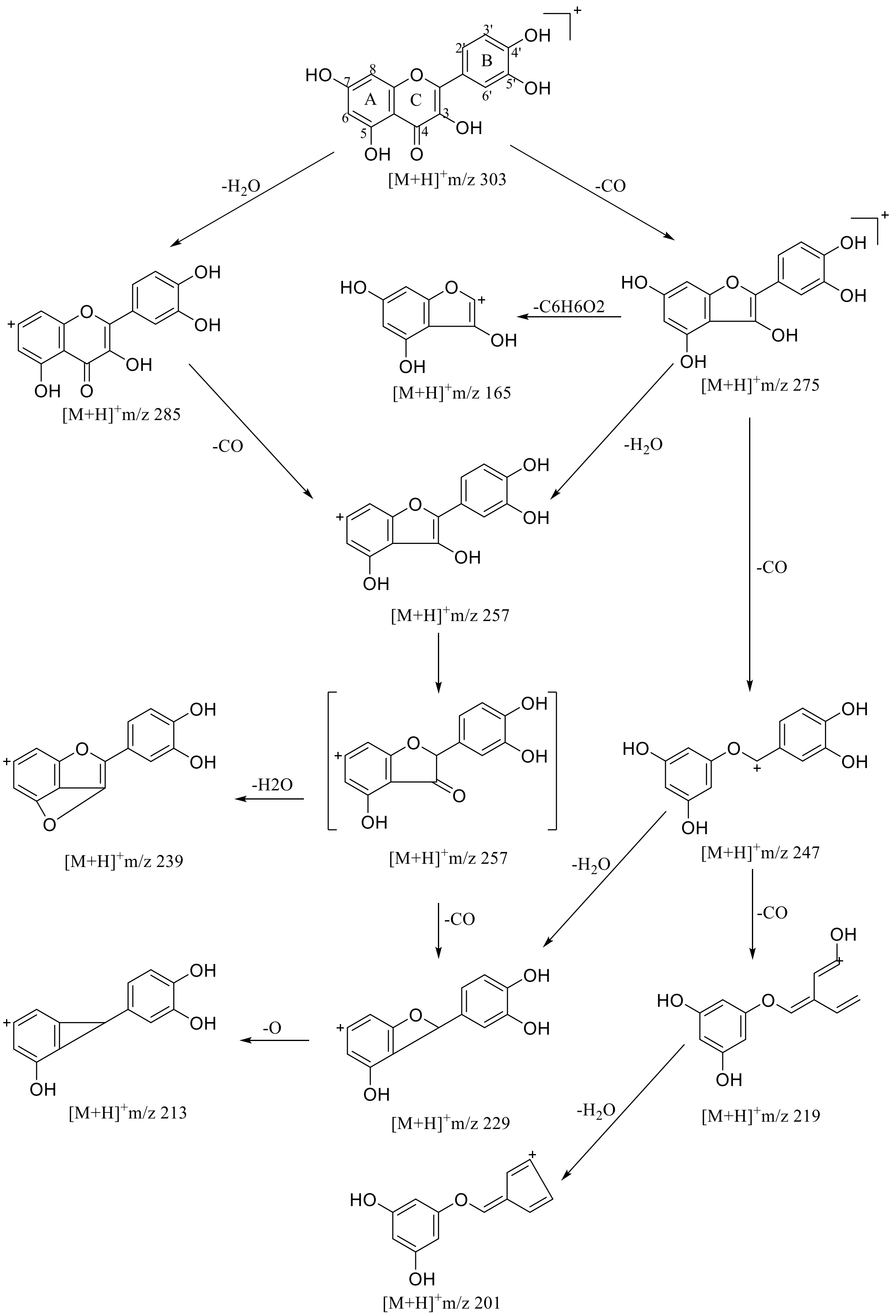

Figure S2. Fragmentation pathways of quercetin
